# Evaluation of the effect of IL-36γ expression on chronic periodontitis by enhancing the MAPK and TLR4 signaling pathways: A basic research

**DOI:** 10.15171/joddd.2018.025

**Published:** 2018-09-18

**Authors:** Amir Reza Babaloo, Adileh Shirmohammadi, Siamak Sandoghchian, Ashkan Kamalzadeh, Shima Ghasemi

**Affiliations:** ^1^Oral and Periodontal Research Center, Tabriz University of Medical Sciences, Tabriz, Iran; ^2^Department of Periodontics, Faculty of Dentistry, Tabriz University of Medical Sciences, Tabriz, Iran; ^3^Department of Immunology, Faculty of Medicine, Tabriz University of Medical Sciences, Tabriz, Iran; ^4^Private Practice, Shiraz, Iran; ^5^Department of Prosthodontics, Faculty of Dentistry, Tabriz University of Medical Sciences, Tabriz, Iran

**Keywords:** ELISA, immunofluorescence, periodontitis, western blot

## Abstract

***Background.*** Periodontitis is an infectious and inflammatory disease of the supporting tissues of the tooth caused by specific microorganisms or a group of microorganisms and, if not treated, leads to progressive degradation of the supporting tissues and subsequent loss of the teeth affected. The aim of this study was to evaluate the effects of IL-36γ on periodontitis by enhancing the TLR4 and MAPK signaling pathways.

***Methods.*** In this pilot study, 50 patients with generalized moderate-to-severe chronic periodontitis and 50 individuals with healthy periodontium, who were candidates for crown lengthening (CL), were selected based on inclusion criteria. The tissue samples were taken during pocket depth surgery (for the test group) and CL surgery (for the control group). The macrophage cells of the inflammatory tissues were extracted and stimulated by TLR4 proteins in a time-dependent manner; then IL-36γ levels in macrophages were investigated. Data were analyzed using descriptive statistics (means ± standard deviations and frequency percentages). Repeat measurement test was used to compare IL36γ expression in MAPK and TLR4 pathways at different time intervals. ANCOVA was used to compare IL36γ expression at different time intervals between the two pathways. Statistical analysis was performed using SPSS 17 at a significance level of P<0.05.

***Results.*** The results of the current study showed a significant relation between TLR4 and IL-36γ (P<0.001); in tissues with generalized moderate-to-severe chronic periodontitis, there was a significant relation between the condition and IL-36γ (P<0.0001). This study also showed that TLR4 and MAPK levels increased in the presence of IL-36γ.

***Conclusion.*** According to the present study, it was concluded that IL-36γ concentrations increased in periodontitis, which could trigger MAPK and TLR4 pathways.

## Introduction

Periodontitis is an infectious and inflammatory disease of the supporting tissues of the tooth,^1,2^ caused by specific microorganisms or a group of microorganisms^1^ and if not treated, it results in progressive destruction of the tooth-supporting tissues and subsequent loss of teeth.^2^ Major tissue destruction in periodontitis is due to unregulated and increased production of inflammatory mediators and degrading enzymes in response to subgingival bacterial plaque.^1^ Inflammatory cytokines play a major role in inflammation; cytokines are soluble proteins used as messengers to transmit signals from one cell to another.^1^ The interleukin-1 (IL-1) family is one of the special cytokines. The members of the IL-1 family, as the central intrinsic and acquired immune mediators, play a key role in the biology of inflammatory diseases. So far 11 members of the IL-1 family have been identified, including 7 ligands with agonist activity (IL-1α, IL-1β, IL-18, IL-33, IL-36α, IL-36β, IL-36g). The members of the IL-1 family include 3 sub-families of IL-1, IL-36, and IL-18.^3^ IL-36 exhibits pro-inflammatory effects and induces the Th17 cytokines.^4^ The subfamily of the IL-36 includes IL-36α, IL-36β and IL-36γ,^3^ which were previously called IL-1F6, IL-1F8, IL-1F9, respectively.^5^ IL-36γ induces inflammatory responses by dendritic cells derived from human monocytes and macrophages, which include the expression of Th17 chemokine.^6^ Lipopolysaccharides (LPS) derived from *E. coli* or *P. gingivalis* (an important bacteria in the etiology of periodontitis)^1^ significantly induce IL-36γ expression in THP-1 ( a human monocytic cell line derived from acute monocytic leukemia patients), which is not the case for IL-36α or IL-36β.^5^ The above-mentioned cases indicate the role of IL-36γ in inflammation, but the mechanism of the effect of IL-36γ has not yet been studied in the oral inflammation. TLR4 (Toll like Receptor 4) is one of the pathogen-specific recognition receptors, which recognizes lipopolysaccharides of the gram-negative bacteria, some of the protected fungal structures of *Mycobacterium* pathogens and some of the internal ligands.^7^ Studies show that TLR4 can increase the expression of IL-36g.^8^ Therefore, in this study we decided to determine IL-36g expression by extracting inflammatory cells such as macrophages from inflammatory tissues of the gingiva (the tissues suffering from chronic periodontitis), and also by inhibition of IL-36g and determining the TLR4 and MAPK pathways, detect the mechanism of the effect of IL-36g on chronic periodontitis to elucidate how IL-36g increases the inflammatory effect. To the best of our knowledge, this is the first such study on chronic periodontitis. Therefore, the present study aims to detect the effect of IL-36g on chronic periodontitis.

## Methods

### Inclusion criteria

The patients in the present study were 25‒70 years of age, with at least 12 teeth (no third molars and no teeth with orthodontic appliances, bridges, crowns and implants), with generalized moderate-to-severe chronic periodontitis, with 30% of the teeth present , with ≥3 mm of clinical attachment loss and radiographic signs of bone loss. The type of surgical packet therapy was resective. The patients were systemically healthy and had informed consent to participate in the study.

### Exclusion criteria

The exclusion criteria consisted of systemic diseases (such as diabetes mellitus, cancers, AIDS, metabolic bone diseases), diseases retarding wound healing, a history of radiotherapy or immunosuppressive treatments, autoimmune diseases, allergies and other infectious diseases, pregnancy or lactation, a history of antibiotic use in the last two months, a history of constant NSAIDs use, and a history of scaling and root planing in the previous year.

### Sample size

Considering that similar studies were not available, a pilot study was used to determine the sample size. The results of the pilot study on 5 candidates detected 64×10^4^ cells per sample and considering that 6400×10^4^ cells were needed, 100 samples were selected, which included 50 subjects with generalized moderate-to-severe chronic periodontitis, who were candidates for removal of or reducing the pocket depth surgery (test group) and 50 periodontally healthy subjects who were candidates for crown lengthening (CL) (control group).

### Methods

Tissue samples were obtained from patients with generalized moderate-to-severe chronic periodontitis during the surgery for removing or reducing pocket depth using submarginal flap and for CL by resective surgery, by considering that we had at least 2 mm of keratinized tissue around the teeth after CL surgery. Considering the fact that the minimum dimensions required for tissue sampling was 3×1 mm, in cases in which the depth of the pocket was ≥5 mm, the sample tissues of submarginal flap were prepared and sent to the immunological laboratory in normal saline solution.

### Stimulation of cells

The cells were stimulated by TLR4 proteins at 2-, 4-, 8-, 12- and 24-hour intervals in a time-dependent manner; then cellular mRNA was extracted by TRIzol (Invitrogen), using real-time PCR and the expression of IL-36γ in macrophages was investigated. Β-actin was selected as a control. Also, some part of the tissues was studied directly with real-time PCR technique using IL-36γ and β-actin primers.

### Isolation and culture of macrophage cells

Macrophages were isolated from generalized chronic periodontitis (GCP) tissues and the tissues were kept directly on ice after resection. The isolation procedure started within 15 minutes. The specimens were weighed and cut into small tissue fragments (1‒2 mm) in Gey’s balanced salt solution (GBSS). The tissues were thereafter incubated in 75 mL GBSS with 0.025 g of collagenase type-1 on a magnetic stirrer at 37°C with continuous pH of 7. After 10 minutes, the suspension and the remaining fragments were filtered through a piece of gauze (pore 60 pm). The suspension was centrifuged at 100 g for 5 minutes and washed twice by suspending the pellet in 50 mL of GBSS and 0.8 pg/mL of DNase. Subsequent to washing, the pellets were suspended in 5 mL of GBSS and 0.8 pg/mL of DNase. The non-parenchymal cells were separated from nonviable cells; the remaining parenchymal cells and erythrocytes were centrifuged on a 16% Nycodenz (Nycomed AS, Oslo, Norway) gradient for 20 minutes at 300 g and 4°C. The low-density fraction was collected, re-suspended in 10 mL of GBSS and 0.8 pg/mL of DNase, and centrifuged at 100 g for 5 minutes. The final pellets were re-suspended in 5 mL of GBSS and 0.8 pg/mL of DNase. The cells (4×10^6^ cells [isolated from each GCP tissue] /well in a 6-well plate) were spread on tissue culture plates at 37°C for 30 minutes before washing and incubating in tissue culture media (DMEM media) containing 5% FBS overnight. Approximately 53% and 38% of these cells were macrophages in GCP tissues and normal controls, respectively, as estimated by their ability to ingest latex beads. Macrophages were isolated separately from each GCP tissue and we selected tissues with at least 4×10^6^ cells. Thirty wells for cases and controls (each well with 8×10^6^ cells) were used per experiment. The experiments were carried out with macrophages for one GCP tissue in each well. The other cells were other phagocytes and lymphocytes. Cell viability was always 90% + SD as assessed by trypan blue. All the experiments were subsequently performed after washing the cells three times with serum-free media. For each experiment, the macrophages were isolated from GCP and normal tissues. The cells were cultured in RPMI1640 cell culture buffer.

### Immunofluorescence

In order to confirm inflammation, after paraffinization of the cells, the specimens were placed in a blocking buffer for an hour and the anti-IL-17 antibody and anti-CD4 antibodies were placed at room temperature for 2 hours for the samples. After washing, the secondary antibody was added for one hour and then, after incubating, the amount of 50 μL of hochest was poured onto the slides and examined under an immunofluorescence microscope.

### RNA isolation and quantitative real-time PCR

The expression of TLR2 and TLR4 was measured by quantitative real-time PCR (qRT-PCR) polymerase chain reaction (qRT-PCR), and all the samples were calibrated by b-actin. Briefly, total RNA was isolated from macrophages using the TRIzol (Invitrogen) isolation solution according to manufacturer’s instructions. Isolated RNA was eluted in RNase-free water and reverse-transcribed with Rever Tra Ace RT-qPCR kit (TOYOBO, Osaka Boseki, Japan). The TLR2 and TLR4 mRNA levels were quantified by qRT-PCR amplification using a 7500 Fast real-time PCR system (Applied Biosystems, Foster, CA, USA) in a total volume of 10 μL, containing 5 μL of SYBR Green 1 mix (Bio-Rad, Hercules, CA, USA), 0.4 μL of forward and reverse primers and 0.06 μL of Tag polymerase, 2.5 μL of ddH20 and 2 μL of cDNA templates. The recommended cycling conditions for qRT-PCR were as follows: denaturation at 94°C for 2 minutes, followed by 35 cycles at 94°C for 10 seconds, at 60°C for 15 seconds, and at 72°C for 30 seconds. The specificity of the amplification products was controlled using a melting curve analysis. The copy number of the objective gene or b-actin transcript in the samples was calculated with the BIO-SYSTEM software program according to corresponding standard curves. Our designed primers were as follows: TLR4 forward primer, 5ʺ-ttgtgcaaacttgccgggagga-3ʺ, TLR4 reverse primer, 5ʺ-acttctccttcagcttggcagc-3ʺ, TLR2 forward primer, 5ʺ-ttgtcccgtgcaaacttgccggggagga-3ʺ, TLR2 reverse primer, 5ʺ-aagtcccgttattacttgccggttagga-3ʺ, b-actin forward primer, 5ʺ-taggaatcctgtggcatccatgaaac-3ʺ, b-actin reverse primer, 5ʺ-taaaacgcagctcagtaacagtccg-3ʺ. These primers have exon-exon boundaries; however, our introns were large enough not to interfere with the PCR and not result in a double melting peak. Each gene was amplified in triplicate. The applicant length of ~100 bp was optimal for use with SYBER Green when running qPCR.

### Investing of relationship between IL-36g and TLR4, MAPK (Western blot method)

Macrophages from GCP and normal tissues, which were treated with HMGB1, were lysed and then underwent electrophoresis on 12% SDS-PAGE gels and transferred onto polyscreen PVDF transfer membranes (PVDF; PerkinElmer, USA). The membranes were blocked with 5% (w/v) non-fat dry milk, 1% (v/v) Tween 20 in PBS for 1 hour at room temperature and incubated overnight with commercially available Anti-Nf-KB antibody (1:1000) (Abcam, USA) at 4°C. Detection was performed with electrochemiluminescence (ECL) and the blots were quantified by densitometry, using an image analysis program (Amercontrol Biosciences, USA).

### Statistical analysis

Data were analyzed with descriptive statistics (means ± standard deviations and frequency percentages). Repeated measurement test was used to compare IL36γ expression level in MAPK and TLR4 pathways at different time intervals. ANCOVA was used to compare expression levels between the two pathways at different time intervals. Statistical analysis was performed using SPSS 17. Statistical significance was set at P<0.05.

## Results

**Figure 1:**
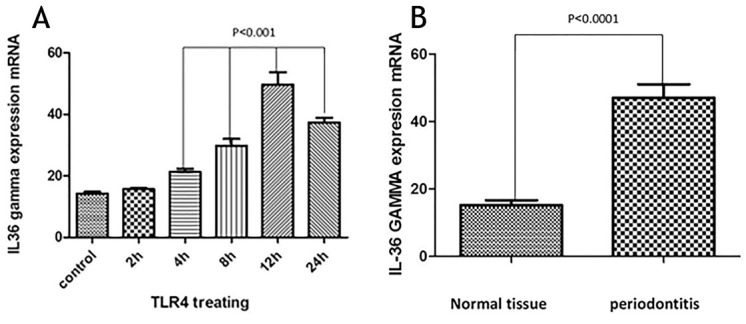
A) IL-36γ expression after treating with TLR4. B) IL-36γ expression.

**Figure 2:**
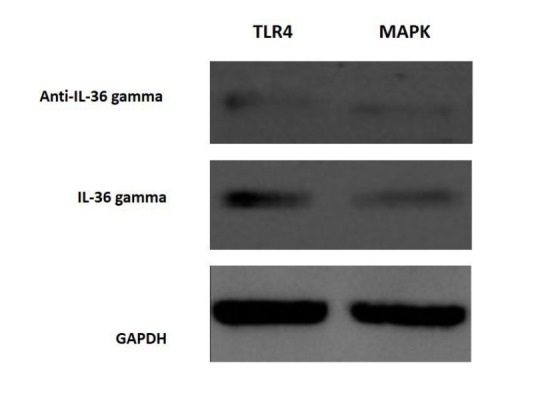
Activation of MAPK and TLR4 pathways.

**Figure 3:**
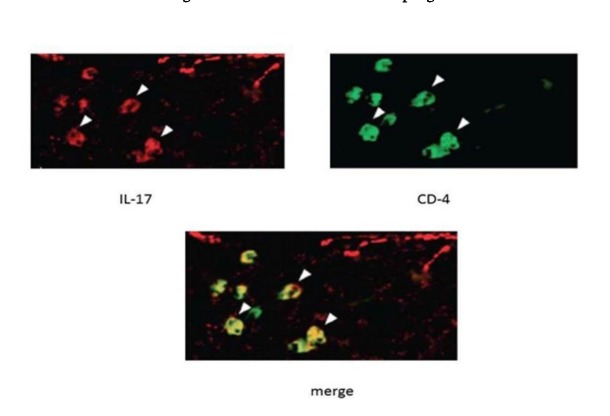
Infiltartion of IL-17 in macrophage cells.

**Figure 4:**
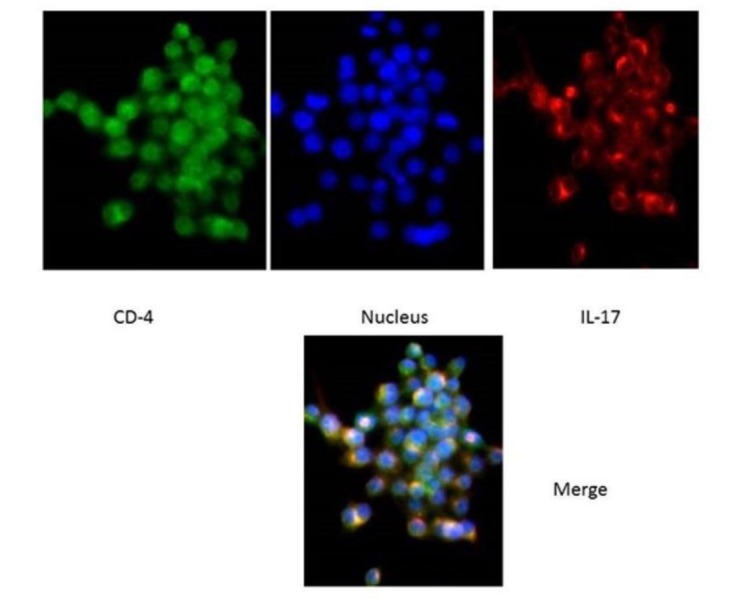
Infiltartion of Th-17 in GCP tissue cells.

The cells were analyzed using IL-36γ polymers after isolation and stimulation with TLR4. The results showed that in the macrophage cells, IL-36γ expression increased by TLR4 stimulation, which was higher with longer duration and reached a peak at 12 hours after stimulation. This indicates a significant relationship between TLR4 and IL-36γ (P<0.001). At 24 hours after stimulation there was a decrease in IL-36γ expression, which might indicate a decrease in the effect of TLR4 on IL-36γ during this interval (Figure 1-a). In order to determine the rate of IL-36γ expression directly in the GCP and normal tissues, the specimens were checked directly with real-time PCR by IL-36γ primer; the results showed a significant relationship between GCP tissues and IL-36γ (P<0.0001) (Figure 1-b). To evaluate the relationship between IL-36γ and TLR4 and MAPK, western blot technique was used to determine protein levels. The results showed that the TLR4 and MAPK levels increased in the presence of IL-36γ, but by using anti-IL-36γ that inhibited IL-36γ the TLR4 and MAPK levels decreased, which was less in TLR4 (Figure 2), possibly due to the fact that gram-negative bacteria are attached to the TLR4 receptors by their LPS and stimulate these receptors. To investigate the infiltration of inflammatory cytokines and to demonstrate the mechanism of inflammation in GCP, anti-CD4 and IL-17 antibodies were used by immunofluorescence method. The results showed that the Th17 penetration rate in the GCP tissues was higher than the normal tissues. This process was performed in the cell culture and the results were compared (Figures 3 and 4).

## Discussion

In the current study macrophages were extracted from tissue samples of patients with GCP and analyzed by isolation and stimulation with TLR4 by IL-36γ polymers. The results showed that in macrophage cells,

expression of IL-36γ increased by TLR4 stimulation, indicating a significant relationship between TLR4 and IL-36γ. In order to determine the amount of IL-36γ directly in the GCP and normal tissues, the tissues were examined directly with the IL-36γ primer, which showed a significant relationship between inflammatory tissues and IL-36γ in GCP tissues (P<0.1000). CD4 and IL-17 antibodies were used to investigate the infiltration of inflammatory cytokines and to demonstrate the mechanism of inflammation in GCP. The results showed that Th17 penetration rate in GCP tissues was higher than that in healthy tissues, indicating inflammation in the affected tissue. Studies have shown that IL-36γ, a member of the IL-36 family, serves as a proinflammatory cytokine in inflammation.^9,10^ One study showed selective IL36γ expression in response to *P. gingivalis* in comparison to IL36α and IL36β.^6^ In one study undertaken to determine the levels of IL-33, IL-36γ and IL-36β in the GCF gingival fluid in different periodontal patients, the group with aggressive and chronic periodontitis exhibited a higher concentration of IL-36β and IL-36γ in GCF compared to the group with gingivitis (P<0.008).^11^ The results of studies showing the role of IL-36γ in inflammation are consistent with those of the present study. The present study showed that IL-36γ could be involved in inflammation of the GCP. One study showed that IL-36γ not only stimulated the expression of the Th17-attractive chemokines and neutrophils (CXCLL/IL-A and CCL20, respectively), induced by dendritic cells and human macrophages, but also it induced the expression by the oral epithelial cells. In addition, the ability of IL-17 to stimulate oral epithelial cells to express IL-36γ makes it possible for these two cytokines to form an inflammatory axis in the oral mucosa.^6^ As the present study showed, the Th17 penetration rate in the affected tissue was higher than that in the normal ones, which could be due to an increase in the IL-36γ expression. One study indicated that macrophages have a high TLR4 expression level and when exposed to bacterial LPS will have proinflammatory cytokine production.^12^ Another study reported that evidence indicates the role of TLR4 in the pathogenesis of periodontal disease,^13^ demonstrating the effect of IL-36γ expression on TLR4 pathway and inflammation. MAPK plays an essential role in various cell types, including proliferation, differentiation, stress response, inflammation, apoptosis and immune defense,^14^ and has an important role in the expression of proinflammatory cytokines.^15^ In this study, we examined the association between IL-36γ and TLR4 and MAPK pathways to evaluate the effect of this cytokine on them; the results showed that TLR4 and MAPK levels increased in the presence of IL-36γ and decreased by IL-36γ inhibitor (anti-IL-36γ). This decrease was less in TLR4, which might be due to the fact that the bacteria present in the oral cavity, which were G-negative, attached to the TLR4 receptors by their LPS and stimulated these receptors. Therefore, the present study showed the effect of IL-36γ on the excitability of TLR4 and MAPK pathways. Overall, our study was unique because it was carried out for the first time, with no study available on these pathways in chronic periodontitis; however, the results of similar studies on the members of the IL-1 family were consistent with the current study.

## Conclusion

According to the present study, it was concluded that IL-36γ increased in periodontitis, which could stimulate MAPK and TLR4 pathways. In order to inhibit the MAPK and TLR4 pathways and its subsequent inflammation, further research is necessary. The results of the current study might lead to preparation of biologic and immunologic medications instead of chemical antiinflammatory medicines.

## Acknowledgments

None

## Authors' Contributions

ARB and ASh carried out surgeries and prepared the samples. SS was responsible for laboratory procedures. AK and SG were responsible for case selection, screening, coordination and preparation of the proposal; in addition, they prepared the manuscript.

## Funding

The study was financially supported by the Dental and Periodontal Research Center of Tabriz University of Medical Sciences.

## Competing Interests

The authors declare no competing interests with regards to the authorship and/or publication of this article.

## Ethics Approval

This study was approved by the Ethics Committee of Tabriz University of Medical Sciences under the code IRCT96980.
